# Supplementary material for: The causal role of circulating inflammatory markers in osteoporosis: a bidirectional Mendelian randomized study
Source: Front Immunol. 2024 Jul 18;15:1412298. doi: 10.3389/fimmu.2024.1412298 (PMC11291241; doi:10.3389/fimmu.2024.1412298)
Supplement: Supplementary file 1 [file DataSheet_1.zip › supplementary material/OSTEOPOROSIS-CIM/GCST90274831.tsv.gz_forest.pdf]

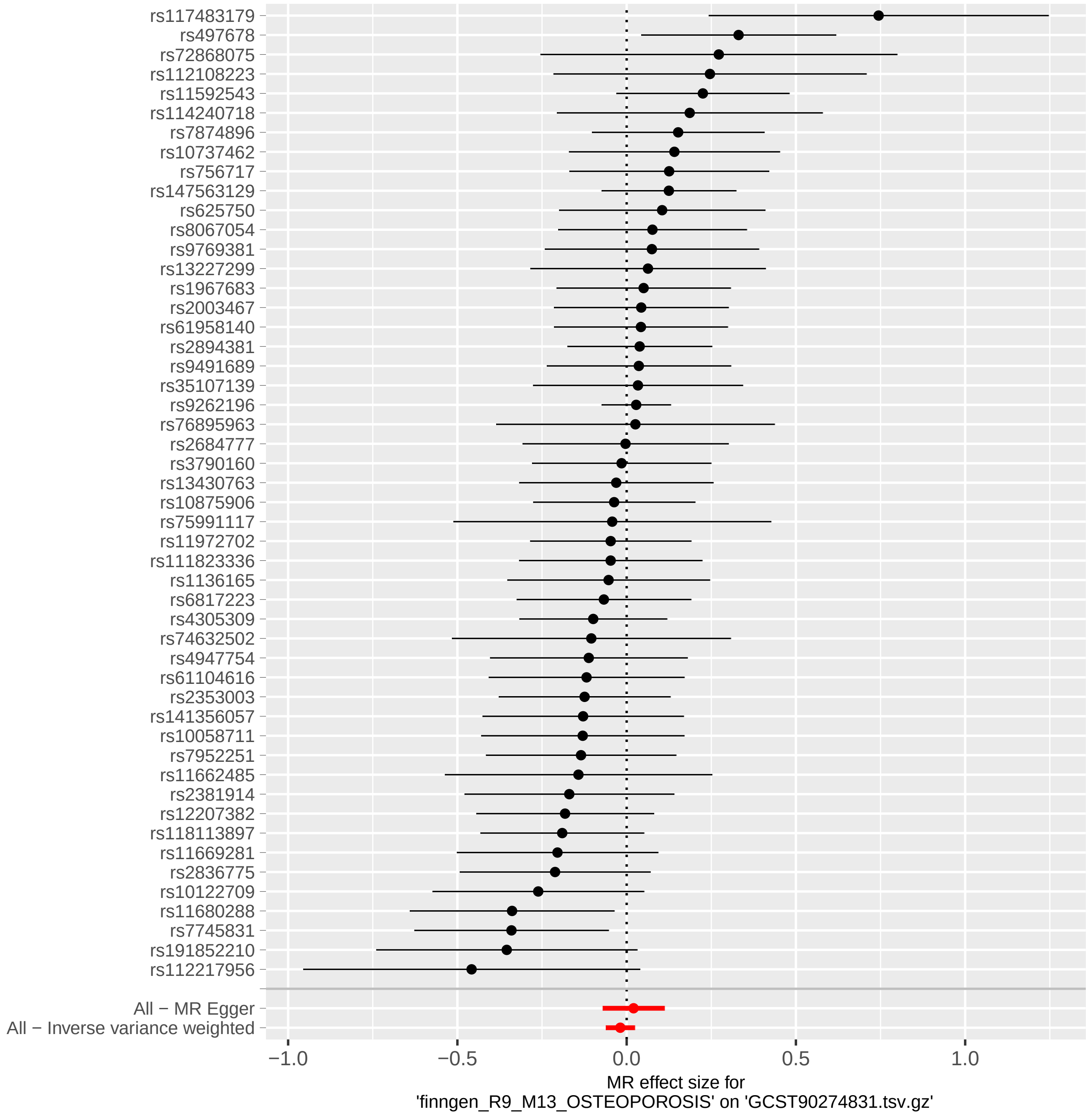

rs117483179
rs497678
rs72868075
rs112108223
rs11592543
rs114240718
rs7874896
rs10737462
rs756717
rs147563129
rs625750
rs8067054
rs9769381
rs13227299
rs1967683
rs2003467
rs61958140
rs2894381
rs9491689
rs35107139
rs9262196
rs76895963
rs2684777
rs3790160
rs13430763
rs10875906
rs75991117
rs11972702
rs111823336
rs1136165
rs6817223
rs4305309
rs74632502
rs4947754
rs61104616
rs2353003
rs141356057
rs10058711
rs7952251
rs11662485
rs2381914
rs12207382
rs118113897
rs11669281
rs2836775
rs10122709
rs11680288
rs7745831
rs191852210
rs112217956
All − MR Egger
All − Inverse variance weighted
−1.0
−0.5
0.0
0.5
1.0
MR effect size for
'finngen_R9_M13_OSTEOPOROSIS' on 'GCST90274831.tsv.gz'
